# Clustering Single-Cell RNA-Seq Data with Regularized Gaussian Graphical Model

**DOI:** 10.3390/genes12020311

**Published:** 2021-02-22

**Authors:** Zhenqiu Liu

**Affiliations:** Department of Public Health Sciences, Pennsylvania State University College of Medicine, 500 University Drive, Hershey, PA 17033, USA; zliu3@phs.psu.edu

**Keywords:** scRNA-seq, cell subpopulation, subspace learning, regularized Gaussian graphical model, parameter-free clustering

## Abstract

Single-cell RNA-seq (scRNA-seq) is a powerful tool to measure the expression patterns of individual cells and discover heterogeneity and functional diversity among cell populations. Due to variability, it is challenging to analyze such data efficiently. Many clustering methods have been developed using at least one free parameter. Different choices for free parameters may lead to substantially different visualizations and clusters. Tuning free parameters is also time consuming. Thus there is need for a simple, robust, and efficient clustering method. In this paper, we propose a new regularized Gaussian graphical clustering (RGGC) method for scRNA-seq data. RGGC is based on high-order (partial) correlations and subspace learning, and is robust over a wide-range of a regularized parameter λ. Therefore, we can simply set λ=2 or λ=log(p) for AIC (Akaike information criterion) or BIC (Bayesian information criterion) without cross-validation. Cell subpopulations are discovered by the Louvain community detection algorithm that determines the number of clusters automatically. There is no free parameter to be tuned with RGGC. When evaluated with simulated and benchmark scRNA-seq data sets against widely used methods, RGGC is computationally efficient and one of the top performers. It can detect inter-sample cell heterogeneity, when applied to glioblastoma scRNA-seq data.

## 1. Introduction

With the advances of single-cell RNA-sequencing (scRNA-seq) technology, large-scale transcriptome profiles of individual cells have been generated at an unprecedented pace. Single-cell gene expression data provide the ability to address a wide-spectrum of biological questions including identifying novel cell subtypes, revealing developmental trajectories, and the study of regulatory mechanisms [1,2]. Efficient computational algorithms are required to take full advantage of these capabilities in complex data. Multiple steps are required for the analysis of scRNA-seq, including quality control, read mapping, expression quantification, normalization, clustering, trajectory discovery and differential gene identification. Unsupervised clustering with scRNA-seq transcriptome is a key step for cell type discovery and distinct cell subpopulation identification, and is critical for novel biological insights [3,4]. Therefore it is especially important to develop reliable and efficient computational methods for scRNA-seq clustering.

The goal of clustering is to group a set of objects into intrinsic subgroups based on their similarities (or distances). This is an important objective of exploratory data mining [5]. There are several different types of clustering algorithms for scRNA-seq. One of the most popular methods is K-means and its variations [6], which iteratively discovers the centers (centroids) of k-clusters and assigns each cell to the closest one through Euclidean distance minimization. Several kmeans based software tools for scRNA-seq data have been developed over the last several years including RaceID [7], RaceID2 [8], SC3 [9], and pcaReduce [10]. The K-means algorithm scales linearly with the number of subjects and is suitable for large datasets. However, it is a greedy algorithm and may only lead to a local optimum. It is sensitive to outliers, biased toward finding equal sized clusters, and may fail to detect rare cell types. The number of clusters, *k*, must be pre-defined, and different values for *k* will lead to different clusters.

Another popular method for scRNA-seq clustering is a graph-based approach, where clusters are detected with community detection algorithms. With scRNA-seq data, a network is first constructed with cells as nodes and cell-cell pairwise distances (similarities) as edge weights; A k-nearest neighbor graph is then built from the network with the k-smallest distances for each cell. Then clusters are discovered by applying a community detection algorithm. While there are several community detection algorithms available, the Louvain algorithm has been widely applied to scRNA-seq [11]. The combination of a k nearest-neighbor graph and a Louvain community detection algorithm for scRNA-seq has led to several software tools including PhenoGraph [12], Seurat [13] and scanpy [14]. The primary advantage of the Louvain algorithm is its speed and scalability. Its time complexity is O(nlog(n)), and the number of clusters does not need to be predefined. However, the number of nearest-neighbors still has to be chosen, and there is no objective method for setting the values of free parameters. There is also evidence that the combination of k nearest neighbor graphs and Louvain does not perform well for smaller datasets [15]. Other approaches for scRNA-seq integrate dimension reduction and cluster methods. Software to accomplish this includes pcaReduce [10], SIMLR [2], SCVIS [16] and VASC [17]. However, these methods require multiple free parameters to be tuned, which is time consuming [18].

Although considerable progress in scRNA-seq clustering has been made over the last several years, clustering remains challenging for scRNA-seq analysis, due to large variability, high dimension, and data loss [19]. Besides the noise in scRNA-seq data, many clustering methods are based on pairwise distance (similarity) of the *k*-nearest neighbors. Due to the high dimension of scRNA-seq data, the distribution of pairwise cell-cell distances becomes concentrated at the average value due to the Law of Large Numbers [20]. Consequently, distances between cells appear quite similar, leading to unreliable clusters. Moreover, there is no consensus on free-parameter determination, and arbitrarily choosing the number of k nearest-neighbors can lead to misleading clustering and visualization.

In this paper, we integrate regularized Gaussian graphical models with a Louvain community detection algorithm. This approach uses high-order(partial) correlation instead of pairwise correlation with multivariable regression. It is robust to noise as a consequence of the regularized method. The regularization parameter λ can be determined by a Bayesian information criteria (BIC) with λ=logp. No free parameter is needed with our proposed method. The adjacency matrix from a regularized Gaussian graphical model is clustered using the Louvain algorithm, and visualized with 2D t-distributed stochastic neighbor embedding (t-SNE). The performance of the clustering can be evaluated with scRNA-seq data with known clusters.

## 2. Methods

Our algorithm integrates a regularized Gaussian graphical model with a Louvain algorithm to cluster and visualize scRNA-seq data. Given a p×n scRNA-seq data matrix $X=[x_{1},x_{2},\ldots ,x_{n}]$, where *p* is the number of genes, and *n* is the number of samples, we cluster the cells into *k* clusters with regularized Gaussian graphical model as described in the following sections.

### 2.1. $L_{2}$-Based Regularized Gaussian Graphical Model

The regularized Gaussian graphical model is related to subspace clustering and Gaussian graphical networks [21,22,23]. Unlike most clustering methods based on pairwise similarity (distance), the proposed method assumes that each cell lies near the union of a low-dimensional subspace, and can be represented by the linear combination of a small number of cells on the same subspace. The problem can be defined as

(1) $$\begin{array}{cc} \min_{W} & ||X-{XW||}_{F}^{2}+{\lambda ||W||}_{F}^{2}\\ \; & \mathrm{diag}(W)=0,\;\;\;\lambda >0. \end{array}$$

where *W* is a n×n adjacency matrix with nonnegative partial (high-order) correlation coefficients $w_{ij}$ as the entrances, λ is the regularized parameter, and ${\left||W|\right|}_{F}=\sqrt{\sum_{ij}w_{ij}^{2}}$. The equation has a decomposable objective function, and is equivalent to *n* multivariable ridge regression problems:

(2) $$\begin{array}{cc} \min_{w_{j}} & \left|\right|x_{j}-Xw_{j}{\left|\right|}_{2}^{2}+\lambda ||w_{j}{\left|\right|}_{2}^{2}\\ w_{jj} & =0,\;\;\;\lambda >0,\;\;\;\forall \;j=1,\ldots ,n, \end{array}$$

which indicates that each $x_{j}$, *j* = 1,…, *n*, is expressed as $x_{j}=Xw_{j}+e_{j}$, $w_{jj}=0$. However, computing each cell individually is time consuming.

Equation (1) without the constraint diag(W)=0 has an analytical solution. Taking the first-order derivative, for the objective function of Equation (1), we have

$$-X^{T}\left(X-XW\right)+\lambda W=0$$

$$\Rightarrow \;\;\;\;W={\left(X^{T}X+\lambda I\right)}^{-1}X^{T}X.$$

To enforce the constraint diag(W)=0, we show that

(3) $$\begin{array}{cc} W & ={\left(X^{T}X+\lambda I\right)}^{-1}X^{T}X={\left(X^{T}X+\lambda I\right)}^{-1}\left(X^{T}X+\lambda I-\lambda I\right)\\ \; & =I-\lambda {\left(X^{T}X+\lambda I\right)}^{-1}=I-\lambda S, \end{array}$$

where $S={\left(X^{T}X+\lambda I\right)}^{-1}$. Therefore, we can estimate *W* after computing $S={\left[S_{ij}\right]}_{n\times n}$ by

$$w_{ij}=\left\{\begin{array}{cc} -\lambda s_{ij} & \mathrm{if}\;\;i\neq j,\\ 0 & \mathrm{if}\;\;i=j. \end{array}\right.$$

By setting diag(S)=0, we have W=−λS, indicating *W* is negatively proportional to the off-diagonal entrances of $S={\left(X^{T}X+\lambda I\right)}^{-1}$. After estimating *W* from *S*, we treat *W* as an adjacency matrix of a network and apply the Louvain algorithm to cluster the cells into subtypes.

The Louvain algorithm aims to partition a network into clusters of strongly correlated cells, while cells belonging to different clusters are weakly correlated. The algorithm clusters cells through modularity function maximization [11]. The modularity function also measures clustering quality and can be used to compare the performance of different clustering methods. Although the Louvain algorithm was originally designed to partition a network adjacency matrix with nonnegative elements, we will generalize it to *W* which may contain negative correlations. Given *W*, the modularity function to be maximized is defined as

$$Q=\frac{1}{2m}\sum_{ij}\left[w_{ij}-\frac{k_{i}k_{j}}{2m}\right]\delta \left(c_{i},c_{j}\right),$$

where $w_{ij}$ is the weight (partial correlation) between cells $c_{i}$ and $c_{j}$, $k_{i}=\sum_{j}w_{ij}$ is the sum of edge weights of cell $c_{i}$, $k_{j}=\sum_{i}w_{ij}$ is the sum of edge weights of cell $c_{j}$, and $\delta (c_{i},c_{j})$ is a indicator function, where $\delta (c_{i},c_{j})=1$, if $c_{i}$ and $c_{j}$ belong to the same cluster, and 0 otherwise. *Q* is the objective function to be maximized. Larger *Q* indicates better cluster separation. The Louvain algorithm determines the number of clusters automatically. The combined regularized Gaussian graphical model and Louvain algorithm is presented in the following steps: Given a scRNA-seq data *X* and a positive λ, (i) first preprocess the scRNA-seq data through transforming the count matrix with log2(X+1), adjusting sequencing depth with quantile normalization, and transforming each cell into zero mean and unit variance; (ii) then calculate $S={\left(X^{T}X+\lambda I\right)}^{-1}$ and normalize $W=-S*\mathrm{diag}{\left(S\right)}^{-1}$ or $W=-\mathrm{diag}{\left(S\right)}^{-\frac{1}{2}}S\mathrm{diag}{\left(S\right)}^{-\frac{1}{2}}$; (iii) and then detect cell clusters with Louvain algorithm by maximizing $Q=\frac{1}{2m}\sum_{ij}\left[w_{ij}-\frac{k_{i}k_{j}}{2m}\right]\delta \left(c_{i},c_{j}\right)$; (iv) output clusters *C* and optimal modularity *Q*.

### 2.2. The Grouping Effects of $L_{2}$-Based Regularization

Given a group of highly correlated cells, $L_{2}$-based ridge regression tends to make the coefficients of highly correlated cells equal, known as the grouping effects of $L_{2}$[24]. Consequently, highly correlated cells may be clustered together in downstream analysis. Sparse regularized regressions (e.g., $L_{1}$/$L_{0}$) are inclined to select only one of the correlated cells randomly with a nonzero coefficient, and do not have the grouping effect. The grouping effects of $L_{2}$-based ridge regression can be shown in the following Lemma:

**Lemma** **1.** *Given a columnwise normalized scRNA-data matrixXwith 0 mean and standard deviation of 1, assuming that*$x_{k}$*and*$x_{l}$*are the expressions of cellskandl, respectively, and let the optimal solution of Equation* (2)* be* ${\bar{w}}_{j}$*, we have*

$$|{\bar{w}}_{jk}-{\bar{w}}_{jl}|\leq \frac{1}{\lambda }\sqrt{2(1-x_{k}^{T}x_{l})}.$$

$x_{k}^{T}x_{l}$ *measures the correlation coefficient between* $x_{k}$ *and* $x_{l}$*. Following Lemma 1,* ${\bar{w}}_{jk}\approx {\bar{w}}_{jl}$ *when* $x_{k}^{T}x_{l}$ *is high (close to 1).*

**Proof of Lemma** **1.** Let

$$E_{j}=\min_{w_{j}}\left|\right|x_{j}-Xw_{j}{\left|\right|}_{2}^{2}+\lambda ||w_{j}{\left|\right|}_{2}^{2}$$

Taking the derivative,

$$\frac{\partial E_{j}}{\partial {\bar{w}}_{jk}}=-2x_{k}^{T}\left(x_{j}-X{\bar{w}}_{j}\right)+2\lambda {\bar{w}}_{jk}=0,$$

and

$$\frac{\partial E_{j}}{\partial {\bar{w}}_{jl}}=-2x_{l}^{T}\left(x_{j}-X{\bar{w}}_{j}\right)+2\lambda {\bar{w}}_{jl}=0.$$

Together we have

$${\bar{w}}_{jk}-{\bar{w}}_{jl}=\frac{1}{\lambda }{\left(x_{k}-x_{l}\right)}^{T}\left(x_{j}-X{\bar{w}}_{j}\right).$$

$$\begin{array}{cc} \Rightarrow \;\;\;|{\bar{w}}_{jk}-{\bar{w}}_{jl}| & =\left|\right|\frac{1}{\lambda }{\left(x_{k}-x_{l}\right)}^{T}\left(x_{j}-X{\bar{w}}_{j}\right){\left|\right|}_{2}\\ \; & \leq \frac{1}{\lambda }\left|\right|x_{k}-x_{l}{\left|\right|}_{2}\left|\right|x_{j}-X{\bar{w}}_{j}{\left|\right|}_{2}. \end{array}$$

Since $\left|\right|x_{j}{\left|\right|}_{2}=\left|\right|x_{k}{\left|\right|}_{2}=\left|\right|x_{l}{\left|\right|}_{2}=1$, we have

$$\left|\right|x_{k}-x_{l}{\left|\right|}_{2}=\sqrt{2(1-x_{k}^{T}x_{l})}$$

and ${\bar{w}}_{j}$ is the optimal solution of $E_{j}$, we have

$$\left|\right|x_{j}-X{\bar{w}}_{j}{\left|\right|}_{2}^{2}+\lambda ||w_{j}{\left|\right|}_{2}^{2}=E_{j}\left({\bar{w}}_{j}\right)\leq E_{j}\left(0\right)=\left|\right|x_{j}{\left|\right|}^{2}=1.$$

So

$$\Rightarrow \;\;\;|{\bar{w}}_{jk}-{\bar{w}}_{jl}|\leq \frac{1}{\lambda }\sqrt{2(1-x_{k}^{T}x_{l})}.$$

Therefore, $L_{2}$-based Gaussian graphical modeling reduces the noises of scRNA-seq data with the regulazation parameter λ, and simultaneously keeps the coefficients of highly correlated cells approximately equal. The grouping effects of $L_{2}$-based methods make the RGGC algorithm an excellent choice for scRNA-seq data clustering.  □

### 2.3. λ Determination and Performance Measures

The regularization parameter λ determines the shrinkage of the coefficients. The larger the tuning parameter λ, the more shrinkage is in the estimated coefficients. However, our experience indicates that the proposed methods are robust over a wide range of λ for clustering. Similar clustering results are achieved with λ from 0.0 to 1000. For convenience, we choose λ=log(p), or λ=2, according to a Bayesian information criteria (BIC) or Akaike information criterion (AIC), respectively. The rationale for choosing a larger λ is that it leads to smaller variance and reduces the noise of scRNA-seq data.

The modularity *Q* can be used to evaluate the performance of different algorithms without knowing the true cluster labels. With known cell-subtypes, normalized mutual information (NMI) and adjusted rand index (ARI) are used to evaluate the consistency between true labels of the cells and detected clusters. Given the true labels $C_{t}$, and detected clusters $C_{d}$, NMI is defined as $\mathrm{NMI}=I\left(C_{t},C_{d}\right)/\sqrt{H\left(C_{t}\right)H\left(C_{d}\right)}$, where $I(C_{t},C_{d})$ is the mutual information, $H(C_{t})$ and $H(C_{d})$ are the entropies of true labels and detected clusters, respectively. Detailed description of the NMI calculation can be found in [25]. NMI values between 0 and 1 measure the concordance between the detected cluster labels and the true labels. A larger NMI indicates higher concordance with truth and better clustering accuracy.

ARI is another popular measure to evaluate the similarity between the true labels and detected clusters [9,19]. Given n cells with *s* true clusters $C_{t}=\left[C_{t1},\ldots ,C_{ti},\ldots ,C_{ts}\right]$ and *r* detected clusters $C_{d}=\left[C_{d1},\ldots ,C_{dj},\ldots ,C_{dr}\right]$, the overlaps between the two partitions can be summarized into a contingency table, in which each entry $n_{ij}=\left|C_{ti}\cap C_{dj}\right|$ denotes the number of elements in common between the two clusters $C_{ti}$ and $C_{dj}$. Then ARI is defined as

ARI=∑ijnij2−[∑iai2∑jbj2]/n212[∑iai2+∑jbj2]−[∑iai2∑jbj2]/n2,

where $a_{i}$ denotes the sum of the *i*th row of the contingency table, $b_{j}$ is the sum of the *j*th column of the contingency table and () is a binomial coefficient. ARI=1 indicates a perfect overlap, while ARI=0 denotes random clustering. The clustering results are visualized with a heatmap and t-distributed stochastic neighbor embedding (tSNE) for 2D visualization [26].

## 3. Results

### 3.1. Benchmark and Simulation Data

The performance of our RGGC algorithm was evaluated with four benchmark datasets. These four datasets span a wide range of cell types with different numbers of subpopulations, representing a broad variety of single-cell data. More specifically, the four scRNA-seq data are: (1) Buettner Data: Embryonic stem cells under different cell cycle stages [27], which includes 182 cells, 8989 genes, and 3 known cell subpopulations; (2) Kolod. Data: Pluripotent cells under different environment conditions [28], which has 704 cells, 10,685 genes, and 3 cell subtypes; (3) Pollen Data: Eleven cell populations including neural cells and blood cells [29], which includes 249 cells, 14,805 genes, and 11 cell subpopulations; and (4) Usoskin Data: Neuronal cells with sensory subtypes [3], which contains 622 cells, 17,772 genes, and 4 cell subpopulations. Cell subtypes in each data were known in advance, and further validated in corresponding studies, providing a validated gold standard for clustering performance evaluation.

The simulated scRNA-seq data with known cluster structures were generated with the splatter R package [30]. We generated three data sets, each consists of 600 cells and 5000 genes, with different degree of cluster separability. We generated simulated data I with 4 clusters, equal relative abundances of 0.25, and differential gene probabilities of 0.05, 0.1, 0.2 and 0.4, respectively. Simulated data II was also generated with 600 cells, 5000 genes, and 4 subpopulations, but with the unequal relative abundances of 0.35, 0.4, 0.15, 0.1, respectively, and the dropout of 2. We generate simulated data III with 600 cells, 5000 genes, and 5 clusters, the unequal relative abundance of 0.15, 0.20, 0.40, 0.15, and 0.10, respectively, the differential gene probabilities of 0.15, 0.1, 0.1, 0.1, and 0.2, respectively, and the dropout of 1.

### 3.2. Performance Evaluation

We ran the RGGC algorithm with the benchmark data using the raw gene expression matrix *X* without any additional information. We first preprocessed the expression matrix *X* with log transformation log2(X+1), normalized the transformed data along the cells with quantile normalization, and then transformed the data of each cell into zero mean and unit variance with z-score. RGGC was then run with different values for λ. The performance was evaluated with NMI and ARI (Table 1).

As demonstrated in Table 1, RGGC achieves the NMI index of at least 0.88, 0.84, 0.93 and 0.90 and ARIs of 0.92, 0.79, 0.87, and 0.98, for the benchmark datasets of Buettner, Kolodziejczyk, Pollen and Usoskin, respectively. The clustering performances of the RGGC algorithm measured by both NMI and ARI are quite robust across a wide range of λ values from 0.00 to 1000. Both the NMI and ARI values are similar with different regularized parameter values for different benchmark datasets. Therefore, we can simply pick a λ without performing a cross validation. We notice that the robustness of the proposed method comes from the Louvain algorithm, in which clusters (communities) are determined mainly by the relative values rather than the magnitudes of the correlations. Although the magnitudes of the (partial) correlations become smaller with larger λs, the relative order of the (partial) correlations remains the same. Hence, the performance of RGGC does not deteriorate significantly. In addition, the covariance matrix may not always be positive definite with λ=0, particularly when the number of cells is larger than the number of genes. Therefore, a positive λ is recommended in practice to guarantee the existence of the matrix inverse.

To further evaluate the performance of RGGC, we compared it with seven other scRNA-seq clustering software packages, including principal component analysis (PCA) together with Kmeans clustering, single-cell interpretation via multi-kernel learning (SIMLR) [2], SC3 [9], RaceID2 [8], Seurat [13], sparse subspace clustering with $L_{1}$ penalty (SSC($L_{1}$)) [21], and SinNLRR [31]. PCA is a baseline method widely used in scRNA-seq clustering and implemented in SC3 [9], while SIMLR, SC3, RaceID2, and Seurat are the known scRNA-seq packages with the best clustering accuracy in the literature. Both SSC($L_{1}$) and SinNLRR are subspace clustering for scRNA-seq similar to RGGC. While SSC($L_{1}$) seeks to minimize the $L_{1}$ regularization, which is equivalent to find the $L_{1}$ inverse of the covariance matrix, SinNLRR minimizes the nuclear norm, which is the sum of all singular values. All packages are evaluated with their default parametric setting, and several packages including SC3, RaceID, Seurat, and SinNLRR require a gene-filtering step before clustering. The number of principal components (PCs) is set to 30 for PCA, and the percentages of variance explained with 30 PCs range from 75% to 84% with different scRNA-seq data. The number of 30 PCs is also a default setting in SC3 and Seurat. In addition, the number of clusters is set to the true number of clusters, if it is required as an input. The computational results of RGGC and other methods with simulation and benchmark data are reported in Table 2 and Table 3, respectively.

As demonstrated in both Table 2 and Table 3, although RGGC does not always achieve the the best performance, it is at least a top 2 or 3 performer with the simulated and real scRNA-seq data, respectively. With the simulation data, RGGC together with SC3, RaceID2, and Seurat achieve the top 3 performance compared to the rest software packages. In contrary, RGGC, SIMLR, and SC3 have the top 3 NMI and ARI values with the real scRNA-seq data. RGGC achieves the best performance with 2 out of 3 simulated data. It has the NMI of 0.97 and ARI of 0.99 for the second simulated data ad the NMI of 0.98 and ARI of 0.99 for the third one. With the real scRNA-seq data, it is the top performer for the Usoskin data with NMI=0.96 and ARI=0.98, and performs the second best and the best for the Buettner data with NMI=0.88 and ARI=0.92. While SC3 performs the best with the highest NMI and ARI values for the Kolodziejczyk and Pollen data, SIMLR is the top performer for the Buettner data with NMI=0.91 and ARI=0.92 (tied to RGGC), respectively.

One interesting finding is that SIMLR is not one of the top three performers with the simulated data, but performs well with the real scRNA-seq data. One reason is that the results with the real data were reported in their original publication with well-tuned parameters. Its performance with the simulated data may be deteriorated with the default parametric setting. In addition, as demonstrated in Table 2, RaceID2 performs well with the first two simulated data but does not detect the subgroups well with the the third one, indicating the importance of parameter-tuning with those packages. RGGC without parameter adjustment and SC3 with ensemble clustering seem to be robust and perform well with both simulated and real scRNA-seq data.

Another interesting comparison is between RGGC and Seurat. Both of them detect clusters with the Louvain algorithm. However, Seurat is based on the PCA dimension reduction (dimension = 30), pairwise distances and a k-nearest neighbor graph, while RGGC is based on high-order partial correlations. For each gene *j*, our proposed approach is equivalent to estimate the coefficients of a multiple regression $x_{j}=Xw_{j}=x_{1}w_{j1}+x_{2}w_{j2}+\ldots +x_{k}w_{jk}+\ldots +x_{p}w_{jp}$ with penalized ridge regression. We estimate $w_{jk}$ (the correlation between gene *j* and gene *k*), while controlling the effects of the rest genes. $w_{jk}$ is a ${\left(p-2\right)}^{th}$ order partial correlation as it includes remaining p−2 genes in the model. In addition, the proposed approach reduced the noise of the expression data with the regulation parameter λ. As demonstrated in Table 2 and Table 3, RGGC outperforms Seurat with both NMI and ARI measurements in all 3 simulated data and 3 out of 4 real datasets including Buettner, Pollen, and Usoskin. In addition, RGGC and Seurat with the Louvain algorithm can detect the number of clusters automatically. RGGC detects the true number of clusters in all 3 simulated data and 2 out of 4 real datasets (not shown in the Tables) including 3 clusters in Buettner and 4 clusters in Usoskin, while Seurat did not find the correct number of clusters for the first simulated data and any of the 4 real datasets, demonstrating the advantages of high-order correlation.

Another interesting comparison is among RGGC, SSC($L_{1}$), and SinNLRR, All three methods perform clustering with high-order partial correlation. While RGGC minimizes the $L_{2}$ norm, which has the group effect as demonstrated at Lemma 1, SSC($L_{1}$) and SinNLRR are based on the $L_{1}$ and nuclear norm optimizations,respectively. As shown in Table 2 and Table 3, RGGC outperforms SSC($L_{1}$) in all simulated and real scRNA-seq datasets, and it also performs better than SinNLRR in all 3 simulated data and 3 out of 4 real datasets evaluated with both NMI and ARI, indicating the importance of the group effects with $L_{2}$ norm.

**Figure 1:**
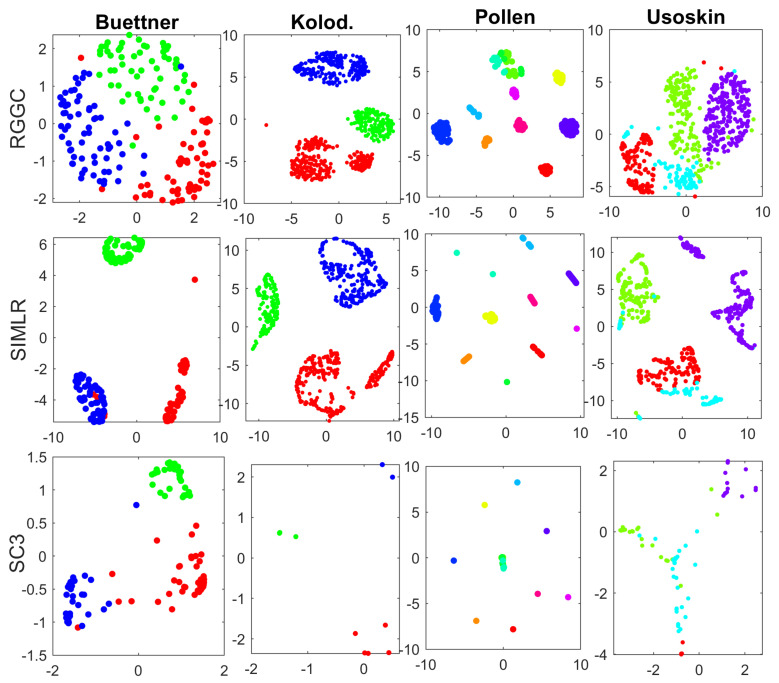
tSNE visualizations with the true clusters of the 4 benchmark data. Similarity (consensus) matrices from RGGC, SIMLR and SC3 are used for tNSE visualization, and different colors represent different true clusters. Top panel: RGGC; Middle panel: SIMLR; Bottom panel: SC3. Datasets to draw the the subplots from left to right: Buettner, Kolodziejczyk, Pollen, Usoskin.

The clusters of the top 3 performers with the real scRNA-seq data including SC3, SIMLR, and RGGC are visualized with tSNE in Figure 1.

Note that the tSNE visualizations of different methods for the same data look quite different in Figure 1. This is because tSNE visualizes each data with the distinct similarity (consensus) matrices from different software. More specifically, each data is visualized with the high-order correlation matrix of RGGC, the kernel matrix of SIMLR, and the consensus (similarity) matrix of SC3, respectively, which can be quite different. We also notice that the number of dots in SC3 looks much less than that in RGGC and SIMLR. In fact, the number of dots represents the number of cells, which must be the same with different methods for each data. SC3 is based on consensus matrix of different clustering algorithms. Therefore, a large number of cells from the same cluster can overlap and stack together, if the clusters from different algorithms are highly consensual. In an extreme case, if all clustering algorithms discover exactly the same clusters, then tSNE will visualize each cluster as a single dot with the consensus matrix of SC3, as demonstrated with the Pollen data of Figure 1.

Figure 1 demonstrates that all three packages are able to separate the true clusters to some extent. SC3 based on consensus clustering separates the true clusters well in two datasets including Kolodziejczyk and Pollen, while SIMLR visualizes the first 3 datasets well, but fails to distinguish one cluster (sky blue) from the rest with the Usoskin data. RGGC is able to separate the true clusters in all 4 datasets, but with less inter-cluster distances. However, it is well known that tSNE only preserves the local neighborhood structure in the data. The inter-cluster distances with tSNE are meaningless [32]. Therefore, RGGC performs well in visualizing the true clusters compared to SIMLR an SC3. It is also computationally more efficient than SC3 and SIMLR. The computational time of RGCC for Buettner, Kolodziejczyk, Pollen, and Usoskin data are 0.03, 0.24, 0.075, and 0.24 s, respectively, while the corresponding computational time of SIMLR are 1.41, 25.1, 2.74 and 14.7 s, and the computational time of SC3 are 28.39, 86.71, 42.69, and 97.61 s on a PC with 12 GB RAM. Finally, it is worth noting that these comparisons are not completely fair to RGGC, as it detects the number of clusters automatically, while most remaining methods use the number of clusters as a known input. However, the number of cell subtypes is usually not known in advance.

### 3.3. Reanalyzing the Glioblastoma scRNA-seq Data

**Figure 2:**
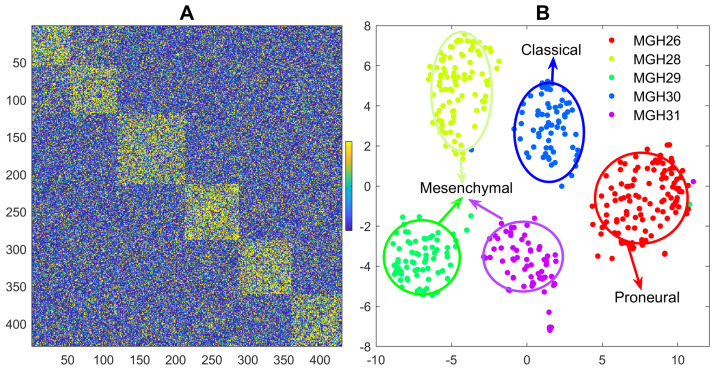
2D visualization with heatmap and tSNE for the detected clusters of five glioblastomas: A: heatmap with the high-order(partial) correlation matrix from RGGC, where orange dots indicate stronger correlation and the numbers with the x- and y- axis represent the cells. The block diagonal (checkerboard) structure of the heatmap indicates well-clustered cells; B: tSNE visualization, where different colors represent different detected clusters. The five well-separated clusters are in line with the tumor samples.

Glioblastoma is a heterogeneous tumor and one of the most lethal human malignancies. An original study by [33] investigated the intratumoral heterogeneity of five primary glioblastomas systematically with scRNA-seq data. Individual cells from five freshly resected and dissociated human glioblastomas were isolated, and the scRNA-seq transcriptomes were generated with SMART-seq. There are roughly 6000 genes and total 430 cells for the five glioblastomas with 96 or 192 cells for each tumor sample. Detailed description of data collection and preprocess with RNA-seq software pipeline are presented in the supplementary material of the original paper. PCA + hierarchical clustering was used to identify 9 clusters with the scRNA-seq data. Clusters detected by RGGC algorithm are shown in Figure 2.

Figure 2 shows that RGGC identifies five distinct clusters along the tumor samples, indicating individual cells from the same tumor are more correlated to each other than cells from different tumors, although heterogeneity exists in the upper left cluster of Figure 2A. The five clusters are highly concordant with the five tumor samples (MGH26, MGH28, MGH29, MGH30 and MGH31) with a NMI of 0.98 and ARI of 0.99. Based on the classification scheme established by the Cancer Genome Atlas(TCGA) [34], the five tumor samples were classified as three glioblastoma subtypes including Proneural (MGH26), Classical (MGH30), and Mesenchymal (MGH28, MGH29 and MGH31) subtypes with population level (buck) expression profiles. Our analysis with individual cells confirms the findings with bulk expression as demonstrated in Figure 2B. Our discoveries with individual cells demonstrate the high inter-sample heterogeneity, but it did not find a strong diversity of transcriptional subtypes within each tumor. Each tumor sample belongs to one glioblastoma subtype. No intratumoral subtype heterogeneity is detected with RGGC, which differs from the original findings. This indicates that different algorithms may lead to different clusters. One reason for the discrepancy is that RGGC with Louvain algorithm detects the number of clusters automatically, while the number of clusters with hierarchical clustering in the original publication was determined heuristically by setting an arbitrary cutoff threshold, which may not always reflect the true data structure.

## 4. Discussion and Conclusions

Clustering is a central component of scRNA-seq data analysis and critical for cell heterogeneity investigations. Downstream analyses are based on identified clusters, and different clusters may lead to different biological conclusions. We propose a regularized Gaussian graphical clustering (RGGC), which is related to subspace clustering. RGGC performs well provided each cell lies in one or multiple low-dimensional subspaces. It discovers the high-order correlation (adjacency) matrix with a $L_{2}$ regularization, which is convex, and the global optimal solution can be found. One main limitation of current clustering methods is that they have free parameters that must be manually defined, and can strongly affect the clustering. Fortunately, RGGC is robust with a wide range of λs, so that we can set λ=2 or log(p) according to AIC or BIC without parameter tuning. Although RGGC does not always perform the best, it is computationally efficient and is one of the top 3 performers with all simulated and real scRNA-seq data. The Louvain algorithm used by RGGC is computationally very scalable, but may stick to a local minimum similar to other clustering algorithms. To overcome this drawback, we recommend to run RGGC multiple times to find the best local optimal solution with the maximal modularity *Q*. Although the proposed algorithm is linear, it is easy to extend it to nonlinear cases with a kernel extension. Note that no single method is always superior with all the datasets. In practice, one way to detect more reliable clusters is to run multiple software packages and identify the consensus clusters.

## Figures and Tables

**Table 1:** RGGC performance measured with NMIs and ARIs with different values of λ for the 4 real scRNA-seq data (detailed information of the 4 data is introduced in Lines 123–132 on page 5), where the regulation parameter λ takes the values from 0 to 1000. NMI and ARI values are reported on the left- and right-sides of the Table, respectively, where higher value indicates better performance.

λ=	NMI				ARI			
	Buettner	Kolod.	Pollen	Usoskin	Buettner	Kolod.	Pollen	Usoskin
0.00	0.88	0.84	0.93	0.96	0.92	0.81	0.91	0.98
0.01	0.88	0.84	0.93	0.90	0.92	0.81	0.91	0.93
0.1	0.88	0.84	0.93	0.96	0.92	0.81	0.91	0.98
1.0	0.88	0.90	0.93	0.96	0.92	0.81	0.91	0.98
2.0 (AIC)	0.90	0.84	0.93	0.96	0.92	0.81	0.91	0.98
log*p* (BIC)	0.88	0.84	0.93	0.96	0.92	0.81	0.91	0.98
2log*p*	0.88	0.84	0.93	0.96	0.92	0.81	0.91	0.98
100	0.88	0.84	0.93	0.96	0.92	0.81	0.91	0.98
1000	0.88	0.83	0.90	0.96	0.92	0.79	0.87	0.98

**Table 2:** Performance comparison of the three simulated scRNA-seq datasets with 8 different clustering algorithms. Detailed information of the 3 simulated data is presented in Lines 133–141 on page 6. Higher NMI and ARI values indicate better performance, and bold numbers indicate the best performance.

Methods	NMI			ARI		
	SimData I	SimData II	SimData III	SimData I	SimData II	SimData III
PCA	0.67	0.70	0.72	0.65	0.70	0.68
RGGC	0.96	**0.97**	**0.98**	0.97	**0.99**	**0.99**
SIMLR	0.74	0.89	0.85	0.71	0.90	0.87
SC3	0.94	93	0.96	0.95	0.95	0.97
RaceID2	**0.97**	**0.97**	0.76	**0.98**	0.98	0.63
Seurat	0.86	0.90	0.94	0.71	0.86	0.93
SSC(L1)	0.72	0.61	0.71	0.64	0.57	0.57
SinNLRR	0.73	0.78	0.83	0.66	0.79	0.81

**Table 3:** Performance comparison of the four real single-cell data sets with 8 different clustering algorithms. Higher NMI and ARI values indicate better performance, and bold numbers indicate the best performance.

Methods	NMI				ARI			
	Buettner	Kolod.	Pollen	Usoskin	Buettner	Kolod.	Pollen	Usoskin
PCA	0.56	0.77	0.83	0.39	0.59	0.75	0.77	0.44
RGGC	0.88	0.84	0.93	**0.96**	**0.92**	0.81	0.91	**0.98**
SIMLR	**0.91**	0.99	0.95	0.74	**0.92**	0.99	0.94	0.68
SC3	0.83	**1.0**	**0.96**	0.87	0.86	**1.0**	**0.95**	0.88
RaceID2	0.31	0.66	0.92	0.35	0.32	0.62	0.91	0.34
Seurat	0.56	0.92	0.90	0.74	0.39	0.90	0.75	0.57
SSC(L1)	0.72	0.64	0.91	0.61	0.78	0.61	0.86	0.55
SinNLRR	0.60	0.78	0.93	0.85	0.63	0.72	0.91	0.88

## Data Availability

The datasets analyzed for this study can be found in the public available scRNA-seq package in R (http://bioconductor.org/packages/release/data/experiment/html/scRNAseq.html, accessed on 15 February 2021). The datasets are also included in our RGGC software in MATLAB. The software RGGC can be downloaded from https://github.com/zliu3/RGGC, accessed on 15 February 2021.
